# Enhanced Methanol Production in Plants Provides Broad Spectrum Insect Resistance

**DOI:** 10.1371/journal.pone.0079664

**Published:** 2013-11-05

**Authors:** Sameer Dixit, Santosh Kumar Upadhyay, Harpal Singh, Om Prakash Sidhu, Praveen Chandra Verma, Chandrashekar K

**Affiliations:** 1 CSIR-National Botanical Research Institute, Council of Scientific and Industrial Research, Lucknow, Uttar Pradesh, India; 2 Academy of Scientific and Innovative Research (AcSIR), Anusandhan Bhawan, 2-Rafi Marg, New Delhi, India; 3 Department of Biotechnology, National Agri-Food Biotechnology Institute, Ministry of Science and Technology, Mohali, Punjab, India; 4 Indian Agricultural Research Institute, Shivaji Nagar, Pune, Maharashtra, India; Centro de Investigación y de Estudios Avanzados, Mexico

## Abstract

Plants naturally emit methanol as volatile organic compound. Methanol is toxic to insect pests; but the quantity produced by most of the plants is not enough to protect them against invading insect pests. In the present study, we demonstrated that the over-expression of pectin methylesterase, derived from *Arabidopsis thaliana* and *Aspergillus niger*, in transgenic tobacco plants enhances methanol production and resistance to polyphagous insect pests. Methanol content in the leaves of transgenic plants was measured using proton nuclear spectroscopy (1H NMR) and spectra showed up to 16 fold higher methanol as compared to control wild type (WT) plants. A maximum of 100 and 85% mortality in chewing insects *Helicoverpa armigera* and *Spodoptera litura* larvae was observed, respectively when fed on transgenic plants leaves. The surviving larvae showed less feeding, severe growth retardation and could not develop into pupae. *In-planta* bioassay on transgenic lines showed up to 99 and 75% reduction in the population multiplication of plant sap sucking pests *Myzus persicae* (aphid) and *Bemisia tabaci* (whitefly), respectively. Most of the phenotypic characters of transgenic plants were similar to WT plants. Confocal microscopy showed no deformities in cellular integrity, structure and density of stomata and trichomes of transgenic plants compared to WT. Pollen germination and tube formation was also not affected in transgenic plants. Cell wall enzyme transcript levels were comparable with WT. This study demonstrated for the first time that methanol emission can be utilized for imparting broad range insect resistance in plants.

## Introduction

Insect pests cause approximately 14% loss in crop productivity and insecticide spray adds to the cost of production, thereby adversely affecting the farm income. Every year ~3 million metric tons of pesticides are used across the globe for pest control with estimated cost of $40 billion [1]. Use of pesticides saves millions of tonnes of food and fibre every year [2], they cause severe environmental and health hazards [3]. Developments in agricultural biotechnology can provide significant new opportunities to address potential challenge of insect pests without causing drastic environmental problems. Genetically modified crops (transgenics) expressing insecticidal proteins like δ-endotoxin of *Bacillus thuringiensis* effectively control several insect pests but they have limited host range. Such transgenic plants have been severely infested by other non target pests in the last few years [4–6]. Hence, there is an urgent need for exploring other possible approaches for imparting broad spectrum insect resistance. Manipulation of plant’s own defence mechanisms may be an approach for achieving broad spectrum insect resistance [7,8].

Plants emit various volatile organic compounds, among which methanol is the second major compound after isoprene [9,10]. Methanol induces defence related genes in *Nicotiana benthamiana* [11]. It also plays vital role in protection of photosynthetic machinery from photo inhibition, stimulating the growth of C3 plants [12], signalling between plants and defence against herbivores [13].

Methanol is produced during de-methylation of cell wall pectin by pectin methylesterases (PME, EC 3.1.1.11). Tobacco cell suspension culture expressing an *Aspergillus niger* PME, showed significant increase in the level of PME and methanol content [14]. Methanol is toxic to insect pests [15]. Herbivore’s attack up-regulates PME activity in several plant species leading to increased emission of methanol. Silencing the expression of PME in plants leads to reduced methanol emission and insect larvae feeding on such plants showed better growth [16].

PME activity and methanol production is highly regulated in plants by multiple mechanisms like- i) differential expression of isoforms in different tissues and at different developmental stages, ii) modifications of local pH in cell wall and iii) simultaneous expression of inhibitory protein (PMEI) [17].

In this study we over-expressed the PME from two different sources *Arabidopsis thaliana* (*AtPME*, accession no. NP_566842) and *Aspergillus niger* (*AnPME*, accession no. XM_001390469) in transgenic tobacco plants independently. Transgenic plants were evaluated for methanol emission and insect resistance against four different polyphagous insects. PME plays critical role in maintaining cell wall structure of plants, therefore, expression of certain important cell wall enzymes and associated structural changes were also analysed. 

## Materials and Methods

### Amplification of PME genes and construction of plant transformation vector

Total RNA was isolated from leaves of *A. thaliana* and mycelium of *A. niger* (Total RNA isolation kit, Sigma, USA) and used for cDNA synthesis (First Strand cDNA Synthesis Kit, Invitrogen, USA). Genes (a*tpme* and *anpme*) were amplified using At-primers [forward primer 5’GCGC**TCTAGA**TAAACAATGGCAACGACTCGAATGGTTAG3’ and reverse primer 5’CTCC**GAGCTC**TCACTTTTGTGGTATAAGCCGAATC3’] and An-primers [forward primer 5’GCGC**TCTAGA**TAAACAATGGTTAAGTCAATTCTTGCATCCG3’ and reverse primer 5’CTCC**GAGCTC**TTTAGTTGATGTAGCTGGTATCAACC3’], respectively. Restriction site of Xba I and Sac I (bold letters in primer sequence) enzyme was also added in forward and reverse primer respectively to assist the cloning process. Amplified genes were directionally cloned between restriction site of Xba I and Sac I enzyme in plant expression vector pBI121. The genes were transcribed under the control of constitutive promoter CaMV35S present in vector backbone. 

### Plant transformation and expression analysis

Expression cassettes were transformed in electro-competent cells of *Agrobacterium tumefaciens* LBA4404. *Agrobacterium*-mediated transformation was performed for the development of transgenic tobacco plants as described earlier [18]. Genomic DNA of different transgenic lines and WT was isolated by DNeasy Plant Mini Kit (Qiagen) and further used for PCR analysis. PCR was performed with gene specific primer of *Atpme* (At-primers) and *Anpme* (An-primers) for the presence of transgene. To analyze the expression of transgene, total RNA was extracted from plant leaves of positive transgenic lines, cDNA synthesized and RT-PCR was performed using gene specific primers. 

### PME Activity Assay

Activity of AtPME and AnPME in transgenic plants was analysed by *in-gel* enzyme assay and titration method [19].

*In-gel* PME assay was performed to analyze the expression of desired PME in transgenic plants. Total soluble protein (TSP) was extracted from transgenic and WT plants in phosphate buffer saline (PBS, pH 7.4). Twenty microgram TSP was mixed with loading dye (without DTT) and separated on 12% SDS-PAGE without heat denaturation. Gel was washed in 2.5% TritonX100 for 5 minutes to remove SDS, followed by PBS, and then incubated with 0.125% citrus pectin solution (prepared in PBS) at 30°C for 45 minutes. Subsequently gel was rinsed in PBS and stained with 0.05% ruthenium red.

Titration method was used to calculate the enrichment of total PME activity in transgenic plants over WT following the earlier described method [19].

### Phenotypic and structural analysis of transgenic plant

Transgenic and WT plants were compared by taking into account phenotypic characters *viz*. Plant height, number of leaves per plant, average leaf area and weight of seed /capsule.

Cell integrity and other structural parameters of selected transgenic plants were analysed by confocal microscopy. Transverse section of top most leaves (one month old transgenic and WT plants) were cut in potato pith. Sections were stained with propidium iodide (Sigma Aldrich, 20µg/ml) for 10 min in dark and examined under confocal microscope (Zeiss LSM510 Meta) at 40X optical zoom with the parameters: excitation, 543nm HeNe1 laser; filter, BP 560-615.

*In* *vitro* germination assay of pollen was performed by germinating the matured pollens on artificial liquid media by hanging drop method. Images were captured after 4 hr of germination by inverted microscope (Nikon DXM 12000F). Further germinated pollens were stained in FDA as per product description and visualized under Confocal microscope (Zeiss LSM 510 Meta) [20].

### Estimation of Methanol in Transgenic plants

#### Quantification of methanol content

Leaf tissue (25 mg) was crushed in phosphate buffer (1ml) prepared in deuterium oxide containing 0.03% (w/v) sodium salt of trimethylsilyl propionic acid (TSP; Sigma-Aldrich) with the help of micro-pestle and then sonicated for 3 mins [21]. Later, sample was centrifuged at 13000 X g for 10 min. Supernatant was collected in a fresh tube and used for further analysis.

The 1H NMR spectra of the polar extracts were recorded at 300 K on Bruker Biospin Avance-III 800 MHz NMR (Bruker GmBH, Germany) spectrometer equipped with a triple resonance cryoprobe. 1D and 2D NMR spectral analyses of aqueous extracts were carried out by dissolving the samples in 500 μl D_2_O and transferring the same to 5mm NMR tubes. A sealed capillary of 20 μl of deuterium oxide containing 0.03% (w/v) sodium salt of trimethylsilyl propionic acid (TSP) was used for quantitative estimation of metabolites and which also served as an internal lock. The 1H NMR spectra with water suppression was obtained using one-dimensional single pulse with 65,536 time domain data points, spectral width of 12,019.23 Hz, a relaxation delay of 5.0 s, acquisition time of 2.72 s, 64 number of scans with 8 dummy scans, free-induction decay resolution 0.36 Hz, a constant receiver gain of 32 with offset frequency set 3771.16 Hz. The 1H NMR spectra of aqueous extracts were manually phased and automated baseline was corrected using TOPSPIN 2.1 (Bruker Analytik, Rheinstetten, Germany). The assignment of methanol was further confirmed by comparing them with the existing literature values and reference compound [21,22]. 

#### Quantification of methanol emission

Stomata on leaf surface are the major site of methanol emission from the plants. Therefore, we quantified methanol content in water emitted through transpiration by leaves surfaces. 

Third leaf of transgenic as well as control plant was detached and placed individually in a polybag in such a manner (with the help of two thin wooden toothpicks) that surface of leaf was not touches to the polybag. Polybag was sealed with the help of hot iron rod (Honda quick poly sealer, India) and hanged in sunlight for at least two hours. Later, polybags were opened slowly and traspirated water was collected from the bottom of polybag with the help of micro-pipette. Methanol was quantified in this transpirated water by Purpald/Alcohol Oxidase method described by Anthon and Barrett, 2004 [23]. Further, experiment was repeated on three biological replicates and the average value was used for calculation. 

### Evaluation of transgenic plants for insect resistance

#### Insect cultures

The larvae of *H. armigera* and *S. litura* were maintained in the laboratory on semi-synthetic diet and castor leaves, respectively. The culture of whitefly (*B. tabaci*) and aphid (*M. persicae*) were maintained separately on tobacco plants. Laboratory was maintained at 26 ± 2°C and 80% relative humidity and bioassays were also performed in similar conditions. 

#### Bioassay against biting and chewing insects (*H. armigera* and *S. Litura*)

Bioassay was performed on detached leaf of one month old transgenic and WT tobacco plants. Leaves were washed in distilled water; air dried and placed in bioassay vials. The neonate larvae of *H. armigera* and *S. litura* were separately released (10 larvae) in each bioassay vial and larval mortality recorded at regular intervals. Three replicates were maintained for bioassay with each insect.

The surviving larvae on transgenic and WT leaves were weighed after 6 days of feeding. The percent growth reduction was calculated by comparing larval weight on transgenic plants with the weight of larvae on WT leaves. 

Best performing T_1_ plants of selected lines (An-4 and At-5) were also tested against the voracious leaf feeder fourth instar larvae of *S. litura*. The larvae were individually released on leaf discs (10 larvae/leaf discs) prepared from selected transgenic and WT plants and observations were recorded for feeding and mortality of larvae. 

#### Evaluation of transgenic plants for efficacy against sap sucking insects (Aphids and Whiteflies)

Transgenic plants were evaluated for resistance against sap sucking insects by *in-planta* bioassay and also using detached leaves. 

#### Aphids

Insect bioassay against aphids was performed by two ways: 1) *In-planta* bioassay 2) Bioassay with detached leaves. In *In-planta* bioassay transgenic plants were evaluated against aphid (*M. persicae*) by releasing them (20 adults/plant) on the youngest leaves of transgenic and WT plants. The number of aphids was counted at regular time interval and percent decrease in aphid population build-up was calculated [24].

Survival of aphid was also analysed on leaf discs prepared from transgenic and WT plants. Aphids were released (10 aphids/ disc) on leaf discs placed on solidified agar (1%) in petriplates and mortality recorded after six days. 

#### Whiteflies

Bioassay against whiteflies was also performed by two methods: 1) *In-planta* bioassay 2) Bioassay with detached leaves. In *In-planta* transgenic plants were evaluated against whiteflies (*B. tabaci*) following the reported protocol [25]. Whiteflies (50 adults/ plant) were released on plants confined within insect proof plastic cylinders covered by fine nylon mesh at top end. Number of whiteflies surviving on each plant was counted after six days.

Survival of whiteflies on transgenic and WT leaf discs was evaluated by following the reported protocol [26] with some modifications. In the modified method, artificial diet in reported protocol was replaced by leaves discs placed on solidified Agar (Figure S1). Agar (1%) was poured in the caps of bioassay vial (2/3 level) and leaf discs were placed on the solidified agar. Leaves placed on solidified agar remained fresh for six days; however we replaced old leaves with fresh leaves on 3^rd^ day of bioassay. Mortality was recorded at regular intervals. 

### Differential gene expression analysis of cell wall enzymes in transgenic tobacco plants

To elucidate the effect of AnPME and AtPME on cell wall enzymes, their transcript level was quantified using gene specific real time primers of various cell wall enzymes as reported earlier [27]. Total RNA was isolated and used for cDNA synthesis as described above. Real-time quantitative PCR was performed with GeneAmp 5700 (Applied Biosystems) using SYBR Green detection dye (Invitrogen). 

### Generation advancement and segregation of transgene in transgenic tobacco plants

Seed of T_0_ transgenic lines of AtPME and AnPME were screened on Kanamycin selection medium (300 mg/l). Putative transgenic seedlings were transferred to pots and kept in glass house conditions for further growth and development (Temperature; 25±2°C, Humidity 60-80%). Seed were harvested after maturation and brought forward to next generation. Best performing T_1_ transgenic lines were further confirmed by genomic DNA PCR, cDNA PCR and PME activity assay, and analysed for insect resistance. 

### Evaluation of fungal and bacterial susceptibility of transgenic plants

Fungus susceptibility of transgenic plants was evaluated against plant pathogenic fungi *Alternaria alternata* and bacteria *Pseudomonas syringae* pv. *maculicola* strain ES4326 (PsmES4326). 

*A. alternata* culture was maintained on potato dextrose agar at 25°C. One week old plate having well developed colony was scraped into 1% gelatine (pre-autoclaved). Number of spores was counted by haemocytometer and stock adjusted to 10^5^ cells/ml. Leaves were surface sterilized and placed on 1% agar plate. Leaves were slightly rubbed by sand paper and 20 µl of spore stock was inoculated through sterile paper disc. Plates were incubated at 30°C for 7 days. Experiment was repeated three times with leaves from three different plants.

A single isolated colony of *P. syringae* was picked and inoculated in 50 ml nutrient broth and allow to grow overnight at 37°C. Bacterial cells were harvested by centrifugation and suspended in 1% gelatine (pre-autoclaved). Bacterial cells were counted by haemocytometer and stock adjusted to 10^5^ cells/ml. Ten spots of 20µl bacterial stock were inoculated on leaves placed on 1% agar plate. Plates were incubated at 30°C for 7 days. Experiment was repeated three times with leaves from three different plants.

### Statistical analysis

All the experiments were conducted in triplicates on biological duplicates. The data was analysed by One way-ANOVA (P <0.05) and means were compared using Duncan’s Multiple Range Test (DMRT) by using SPSS software. 

## Results

### Development of plant transformation vector

**Figure 1:**
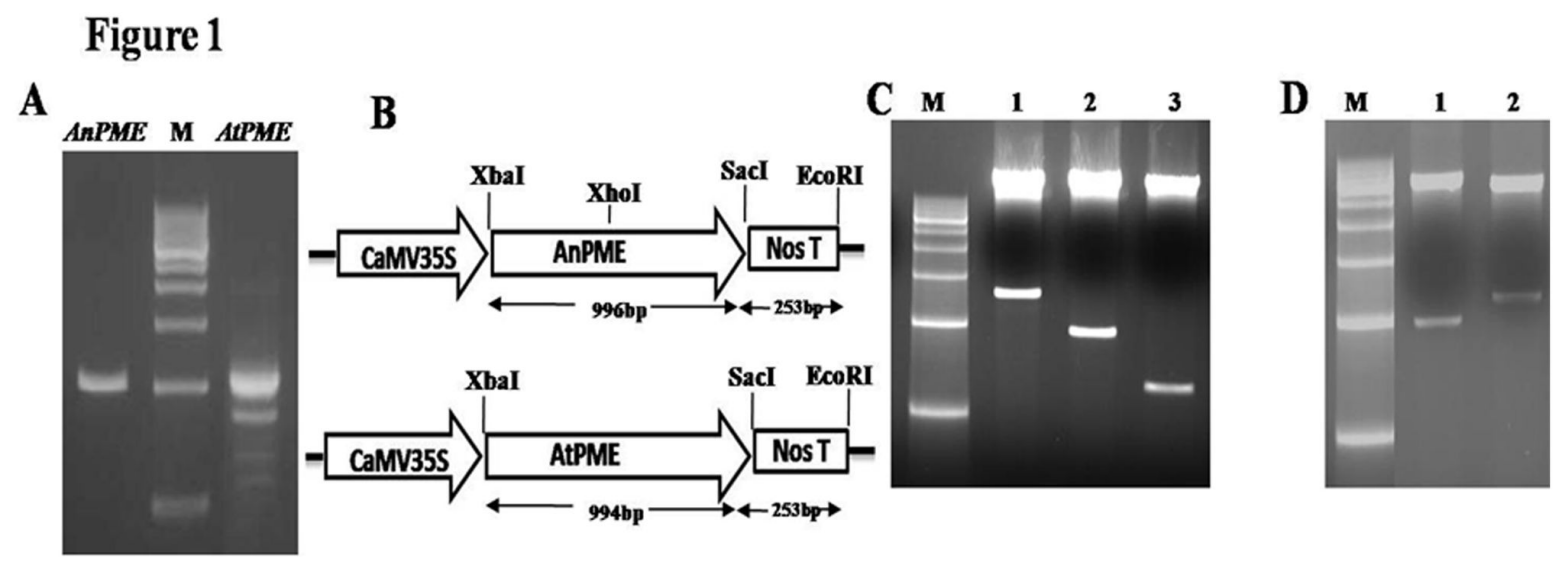
Amplification of PME genes and cloning in plant expression vector. (A) Amplification of *pme* genes, *AnPME*; PME from *A. niger*, M; 500bp DNA ladder, *AtPME*; PME from *A. thaliana*. (B) Schematic representation of the plant expression vector (pAnPME and pAtPME). (C) Restriction analysis of pAnPME ; M, 500bp DNA Ladder ; 1, Digestion with Xba I and EcoRI; 2, Digestion with Xba I and Sac I; 3, Digestion with Xba I and XhoI. D, Restriction analysis of pAtPME; M, 500bp DNA Ladder; 1, Digestion with Xba I and Sac I; 2, Digestion with Xba I and EcoRI.

Total RNA was isolated from *A. thaliana* leaves and *A. niger* mycelia, and cDNA synthesized following the protocols described in materials and methods. Both the genes were amplified by PCR using respective cDNA as template and gene specific primers (Figure 1A). Plant expression cassettes (pAtPME and pAnPME) were developed by cloning the amplified genes in plant expression vector pBI121 (Figure 1B). Cloned genes were confirmed by restriction digestion with different enzymes (Figure 1C and D) and in-frame gene sequences confirmed by sequencing.

### Plant transformation and molecular analysis of putative transgenic lines

**Figure 2:**
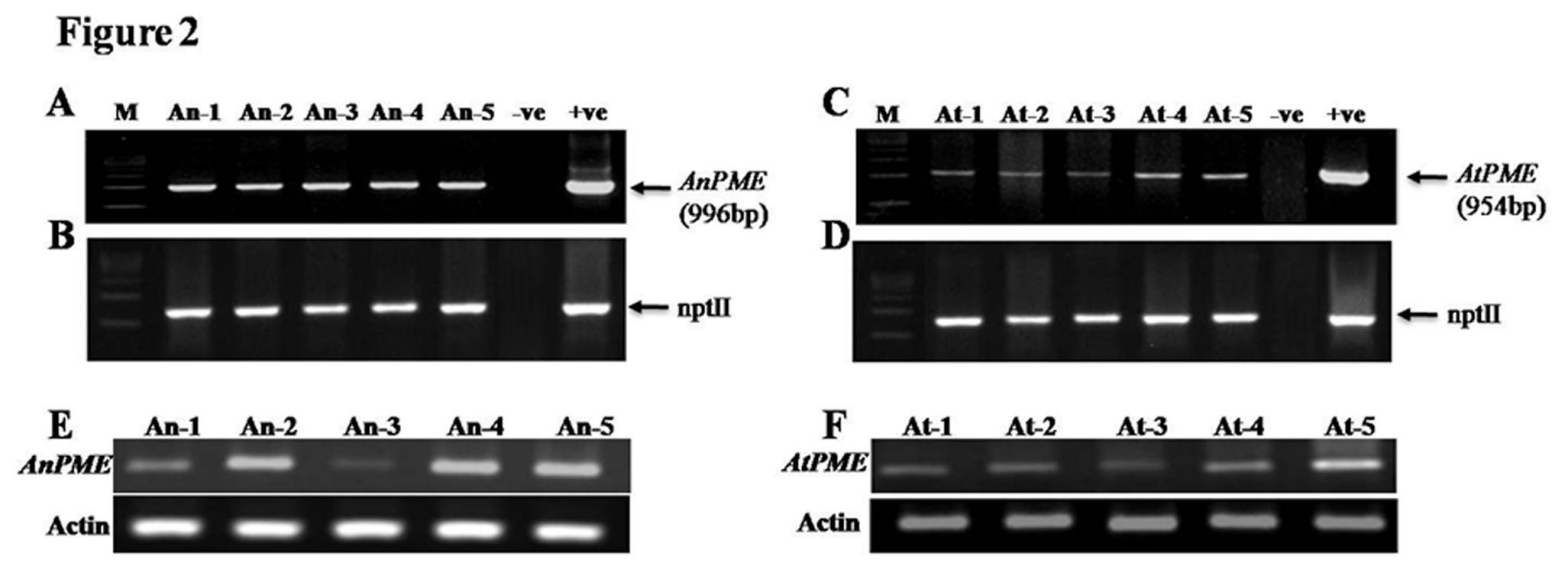
Molecular analyses of putative transgenic lines. Genomic DNA PCR (A) with primer pairs AnF and AnR. (B) with primer pairs nptF and nptR, M; 500bp DNA ladder, An 1-5; different transgenic lines of *AnPME*, -ve; wild type (NTPH), +ve; positive control (pAnPME). (C) with primer pairs AtF and AtR . (D) with primer pairs *nptF* and *nptR*, M; 500bp DNA ladder, At 1-5; different transgenic lines of *AtPME*, -ve; control plant (wild type), +ve; positive control (pAtPME). (E) cDNA PCR of *AnPME* transgenic lines, An 1-5; different transgenic lines of *AnPME*. (F) cDNA PCR of AtPME transgenic lines, At 1-5; different transgenic lines of *AtPME*. Actin was taken as an internal control.

Twenty five transgenic lines were developed for each gene. Integration of genes in transgenic plants was analysed by qualitative PCR with transgene specific primers using genomic DNA as template. Transgenic plants containing *Anpme* was confirmed by using primers AnF and AnR, which amplified 996 bp DNA fragment in PCR (Figure 2A). *Atpme* transgenic plants were analysed using primers AtF and AtR, which amplified 954 bp DNA fragment (Figure 2C). Transgenic plants were further confirmed by amplifying selection marker gene (*nptII*) using primers nptF and nptR, which yielded ~750 bp DNA (Figure 2B and D). PCR amplification with genomic DNA of non transformed wild type (WT) plant did not give any amplification. To analyse the transcript expression of *AtPME* and *AnPME* in transgenic plants, PCR was performed using cDNA as template with above primer sets (Figure 2, E and F). cDNA from WT plants used as negative control did not show any amplification.

### PME activity of transgenic plants

**Figure 3:**
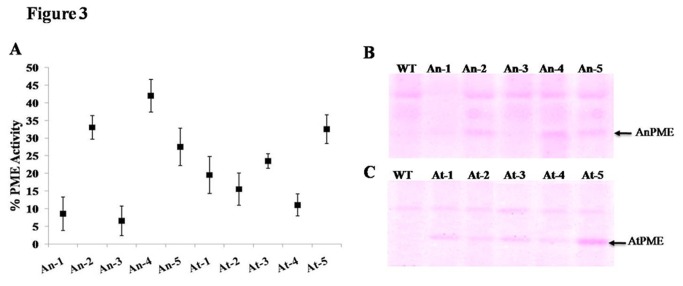
Assay of transgenic plants for PME activity. (A) Percent enrichment of PME activity in different transgenic plants over wild type (WT). (B) & (C) *In-gel* PME activity analysis of different transgenic lines expressing AnPME (An 1-5) & AtPME (At 1-5). Arrow head shows desired PME bands in transgenic plants.

PME activity of transgenic and WT plants was compared by measuring its activity in young leaves of 12 week old plants by titration method and *in-gel* assay. We observed enhance PME activity in both types of transgenic plants in comparison to WT. Transgenic plants of AnPME and AtPME showed up to 42% and 39% increased PME activity, respectively (Figure 3A). Activity was further confirmed by *in-gel* assay. Several PME bands were observed in WT plants which are native PME’s of plants. However, AtPME and AnPME expressing transgenic lines showed an additional PME activity band at ~36 kDa (expected size of both PME), which was absent in WT plants (Figure 3, B and C).

### Estimation of methanol in transgenic plants

#### Quantification of methanol content

**Figure 4:**
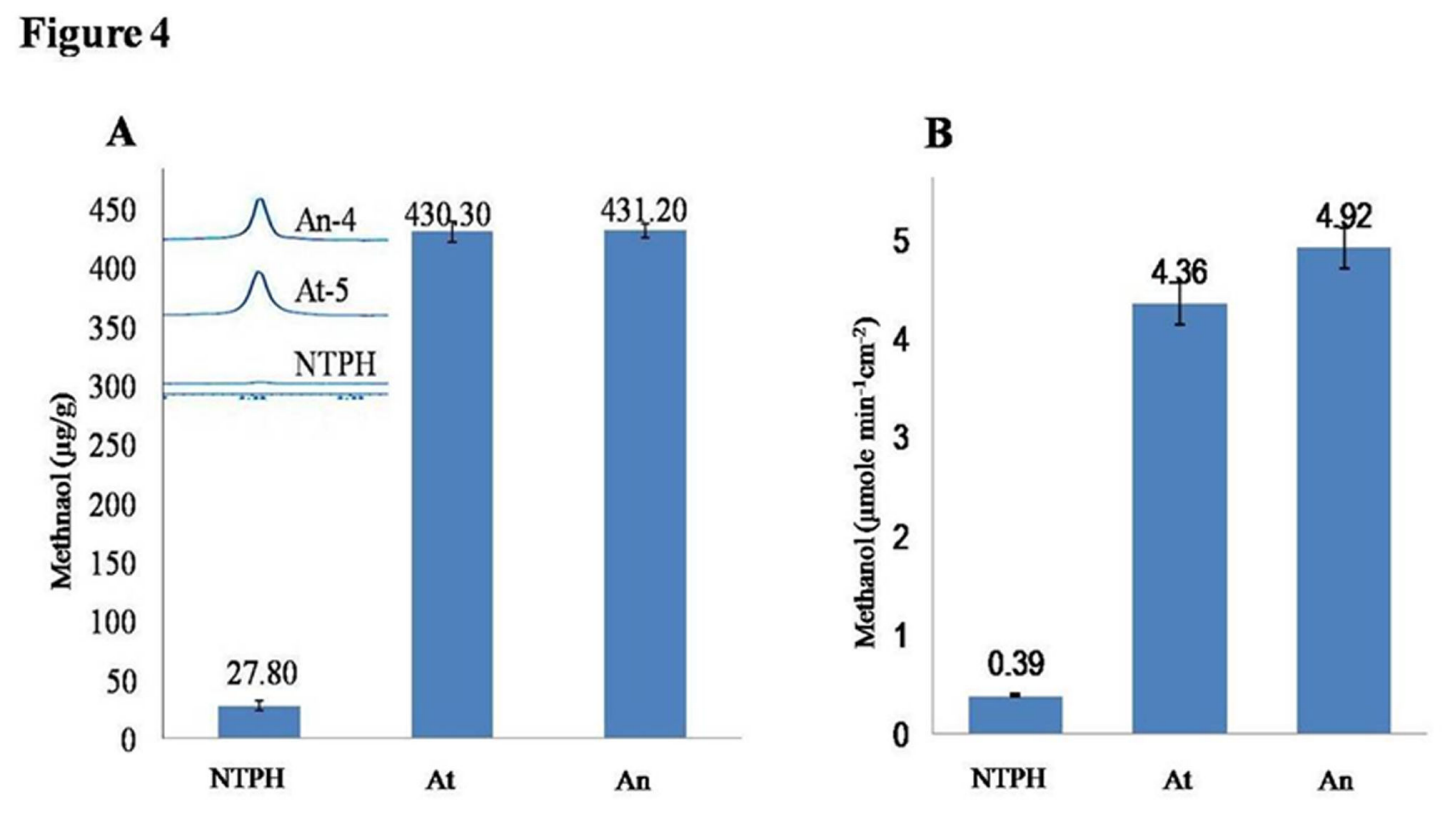
Estimation of methanol in transgenic plants. (A) Quantification of methanol content in control (NTPH) and selected transgenic lines by 1H NMR (inset image shows NMR spectra), Transgenic plants showed up to 16 fold higher methanol content compared to control plants. (B) Quantification of methanol emission in transpirated water through the stomata on leaves surface, Leaves of transgenic plant was also emitted more methanol (~12 fold) than control plant.

PME activity results in methanol production in plants, therefore we analysed the methanol content in the leaves of transgenic and WT plants. Methanol content in the fresh leaf tissue (25 mg) extracts of transgenic and WT plants were measured using 1H NMR spectrum. Transgenic plants showed up to 16 fold higher methanol content as compared to WT plants (Figure 4). We observed ~27.8 µg/g methanol in non transgenic tobacco leaves which increased up to ~432 µg/g in PME over expressing lines. 

#### Quantification of methanol emission

Methanol emission rate from the surface of leaves was quantified in water emitted through transpiration by leaves surface. After two hours of incubation, we collected almost equal volume of water from transgenics as well as control plant leaves containing polybags (table S1). Further, methanol was quantified in this transpirated water by Purpald/Alcohol Oxidase method. Rate of methanol emission of transgenic plants of *AnPME* and *AtPME* were 4.36±0.21 and 4.92±0.21 µmole min^-1^cm^-2^ which were 11.17 and 12.61 fold higher than control plants (0.39±0.01 µmole min^-1^cm^-2^, Figure 4B and Table S1).

### Evaluation of transgenic plants for insect resistance

**Figure 5:**
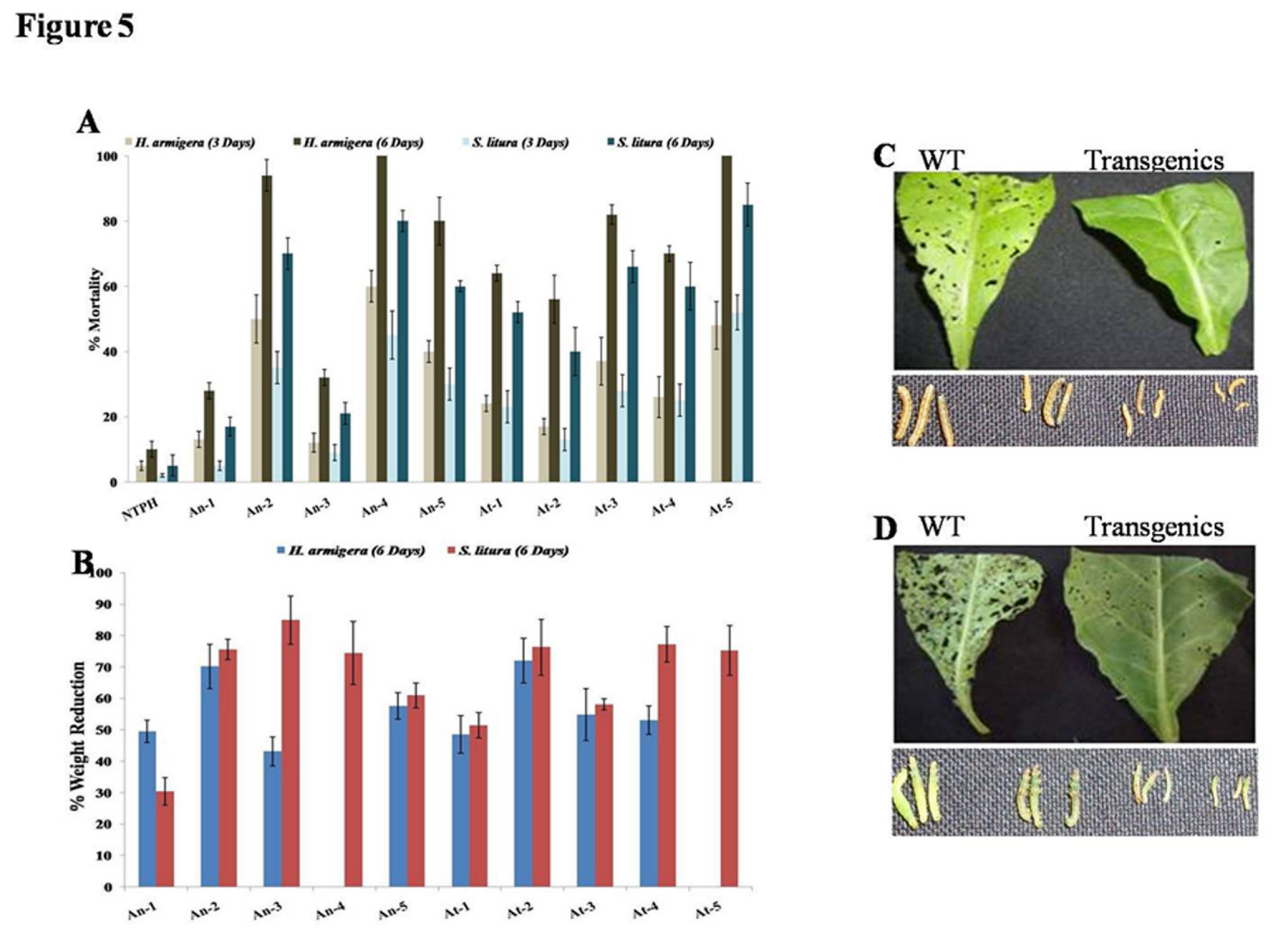
Evaluation of transgenic plants against neonate larvae of *Helicoverpa armigera* and *Spodoptera litura*. (A) Larval mortality on different transgenic lines. (B) Reduction in weight larvae on fed on different transgenic lines. Feeding damage and larval size of *H. armigera* (C) and *S. litura* (D) on wild type and transgenic leaves.

Bioassay against chewing insects was performed on detached leaves. The larvae consumed almost the whole leaf of WT plants and developed normally. However, only small amount of leaf of both *AnPME* and *AtPME* transgenic plants were fed upon larvae. *AnPME* plants caused 12%-60 % mortality of *H. armigera* larvae after 3 days feeding which increased to 28%-100% after 6 days (Figure 5A). Similarly, *AtPME* lines caused 17%-48% and 56%-100% mortality of *H. armigera* larvae after 3 and 6 days of feeding, respectively (Figure 5A). Surviving larvae on both type of transgenic plants showed severe growth retardation (Figure 5B and C). The percentage growth reduction was in the range of 44%-71% and 48%-72% after 6 days on *AtPME* and *AnPME* transgenic plants, respectively (Figure 5B). Feeding of *S. litura* larvae on *AnPME* plant leaves for 3 to 6 days resulted in 5%-45% and 17%-80% mortality, respectively. While the percent mortality on *AtPME* plants leaves was 13%-52% and 40%-85% after 3 and 6 days, respectively (Figure 5A). Weight reduction (%) of surviving larvae on *AnPME* and *AtPME* was 31%-85% and 52%-76% respectively (Figure 5B and D).

Both *AnPME* and *AtPME* transgenic plants showed higher resistance to larvae of *H. armigera* as compared to the larvae of *S. litura*. The best performing lines (line An-4 of *AnPME* and At-5 of *AtPME*) caused 100% mortality of *H. armigera* but could only cause 80%-85% mortality of *S. litura* after 6 days of feeding. However surviving larvae showed severe growth retardation and died eventually. 

Transgenic plants were evaluated for resistance against sap sucking insects by *In-planta* bioassay and further confirmed by leaf disc bioassay. Transgenic plants showed significant resistance against sap sucking insects *M. persicae* and *B. tabaci*.

**Figure 6:**
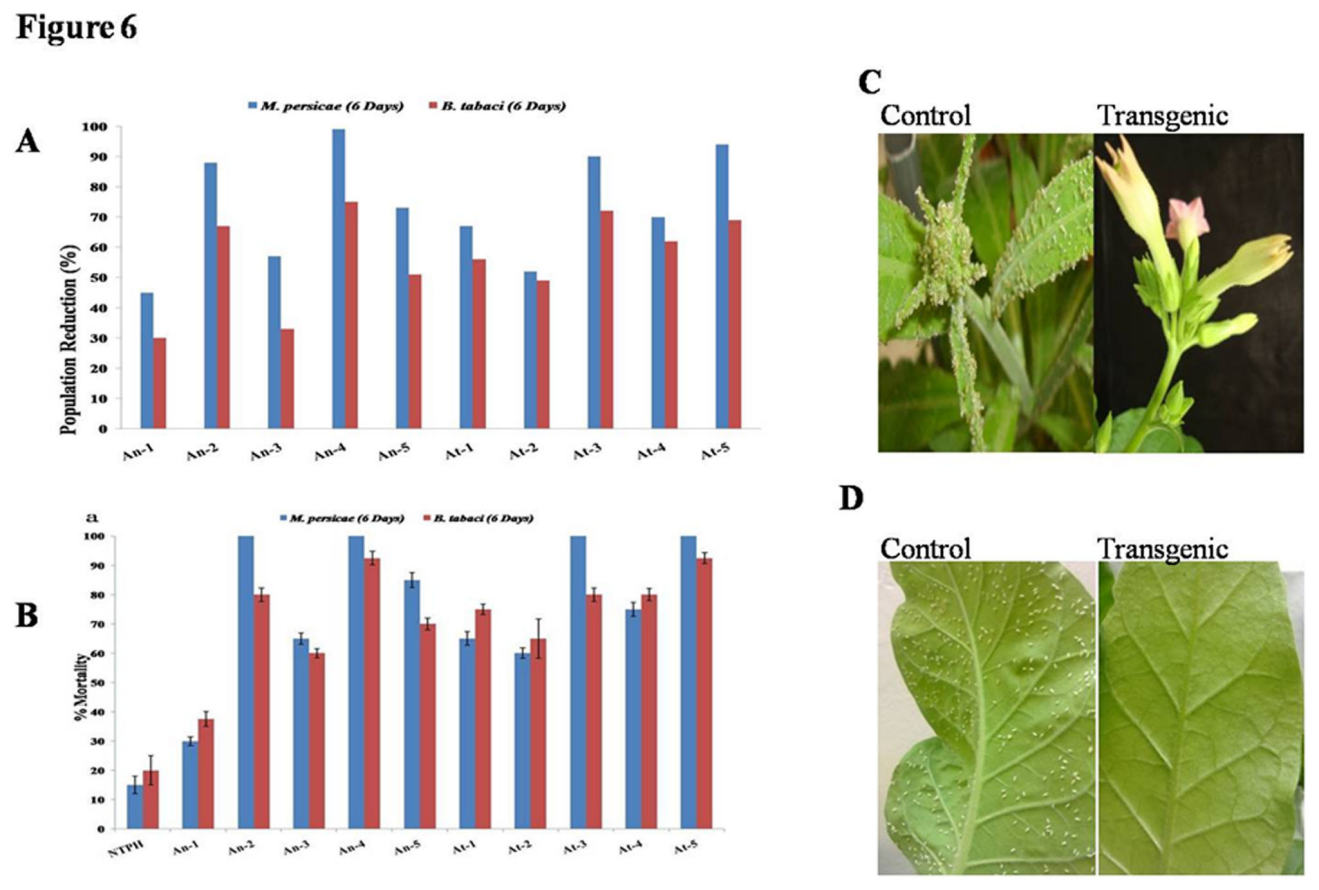
Evaluation of transgenic plants against aphids and whiteflies on different transgenic lines. (A) *In-planta* Bioassay of different transgenic lines with aphids and whiteflies. (B) Bioassay with aphids and whiteflies using detached leaves. Transgenic plant show complete absence of aphids (C) and whiteflies (D).

*In-planta* bioassay against *M. persicae* was performed by releasing insects (20/plant) on youngest leaf of each plant and counting their number after 6 days. Transgenic plants effectively inhibited the multiplication of *M. persicae. AnPME* and *AtPME* transgenic plant caused 45%-99% and 52%-94% reduction in population over control plants, respectively (Figure 6, A and C). In leaf disc assay, *AnPME* and *AtPME* plants caused 30%-100% and 65%-100% mortality of *M. persicae* adults after 6 days, respectively (Figure 6B). *In-planta* bioassay against whiteflies was performed by releasing them on transgenic and WT plants covered by insect proof plastic cylinders and counting their number after 6 days. Reduction in whitefly population after six days on different lines of *AnPME* and *AtPME* was 30%-75% and 49%-72%, respectively (Figure 6, A and D). In leaf disc bioassay *AnPME* plants caused 38%-93% mortality where as *AtPME* plants showed 75%-92.5% mortality (Figure 6B).

### Phenotypic and Structural analysis of transgenic plant

**Figure 7:**
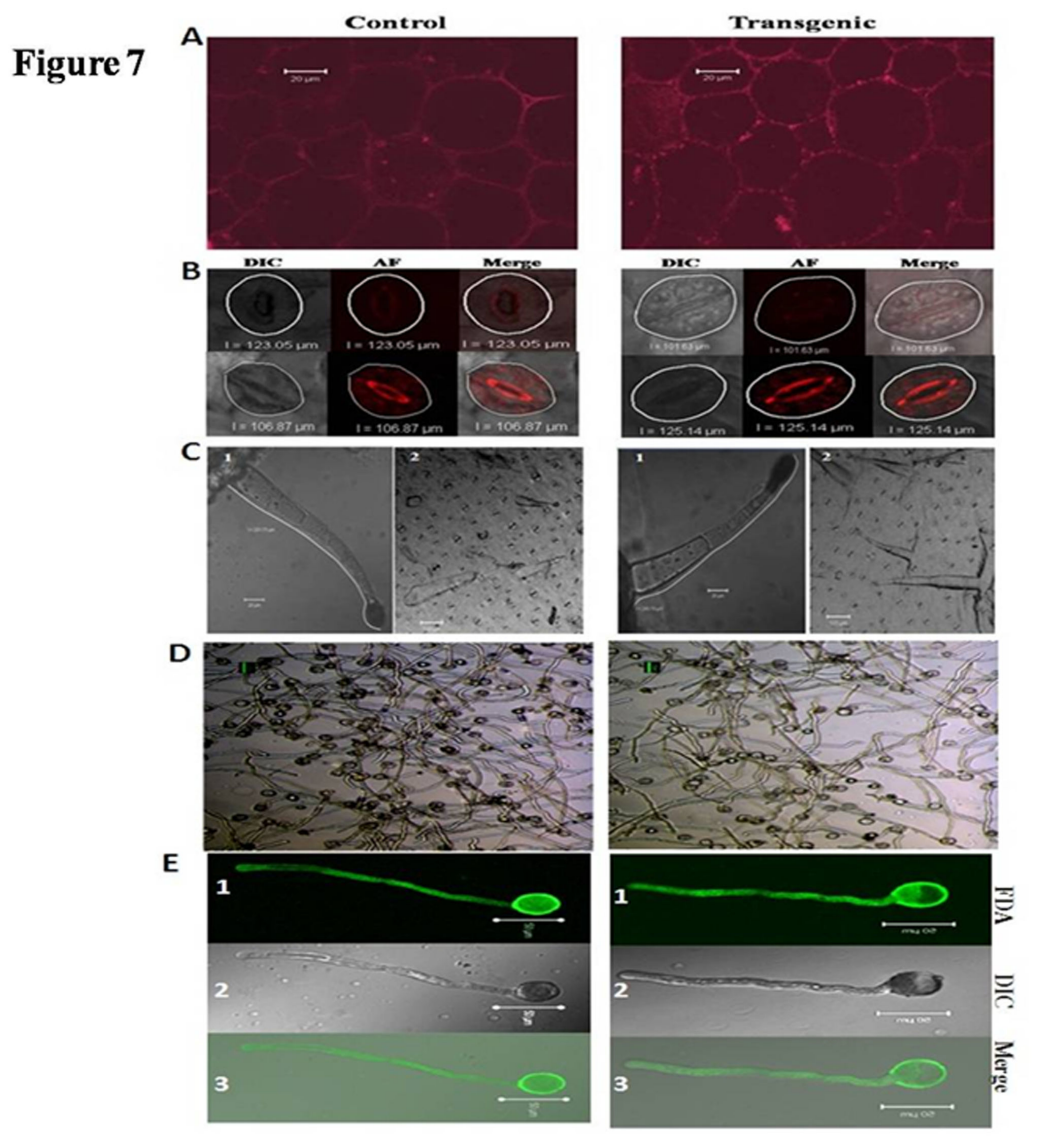
Confocal microscopic images of transgenic and control plants. (A) Propidium iodide stained transverse section of transgenic and control plants showing well integrated cell network. (B) Structure of stomata, circumference of stomata of transgenic plant (113.48±6.7) was similar to WT (114.96±4.6). (C) Structure of trichomes (1) and stomatal density of transgenic and WT plants (2) were also similar to control plant. (D) Pollen germination of transgenic and WT (after 3 hrs). (E) FDA stained single pollen showing equal pollen tube length for transgenic and WT (1). FDA stained (2) DIC and (3) merge of FDA and DIC.

PME plays vital role in plant development hence we have critically examined growth, structural and physiological parameters of transgenic plants. Transgenic plants showed normal growth pattern and morphology as compared to WT plants (Figure S2). Transgenic plants showed well integrated hexagonal cell network as observed in WT plants without any deformities (Figure 7A). Further, we examined structure and density of stomata which was also found similar in transgenic and WT plants (Figure 7B). Number of stomata on lower surface of transgenic plants (95 ± 12 per mm^2^) was almost similar to WT (103 ± 9 per mm^2^). Average circumference of stomata of transgenic plant (113.48 ± 6.7 µm) was also similar to WT (114.96 ± 4.6 µm). Naturally tobacco plants had uniseriate type of trichomes, both transgenic and WT plants showed well organized uniseriate trichomes with well arranged glandular hair (Figure 7C). PME also played critical role in pollen germination and pollen tube formation. We studied pollen viability by pollen germination assay followed by fluorescein diacetate (FDA) staining. *In vitro* pollen germination assay on artificial liquid media showed more than 95% pollen germination for both transgenic and WT plants (Figure 7D). Confocal microscopic images showed almost equal length of pollen tube of transgenic (3.8 ± 0.08 µm) and WT pollens (3.9 ± 0.05 µm) after 4 hrs (Figure 7E).

### Transcript level of important cell wall enzymes in transgenic plants

**Figure 8:**
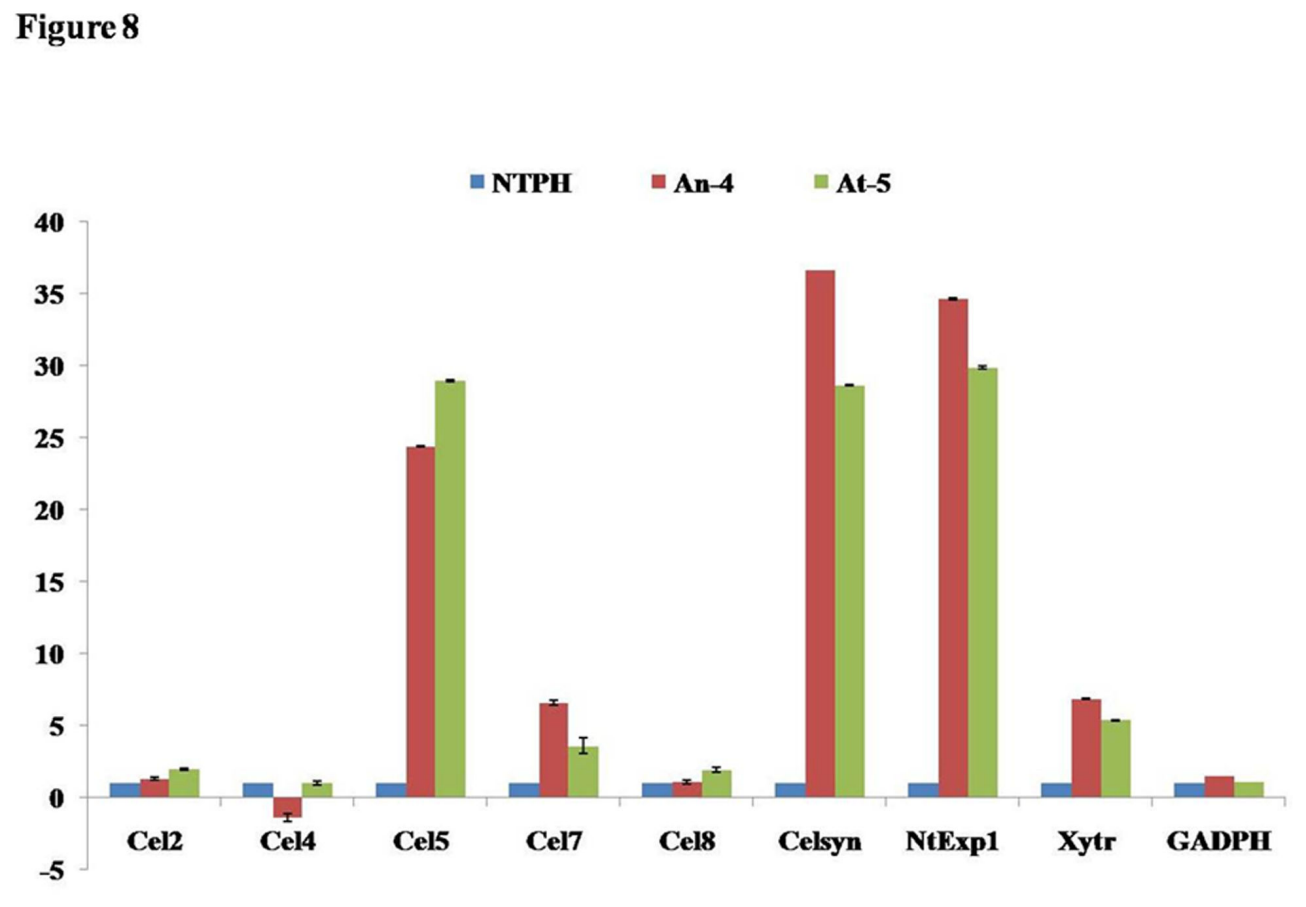
Transcript level of important cell wall enzyme in transgenic plant. Transcript level of all analysed genes [endo-1,4-ß-glucanases (Cel2, Cel4, Cel5, Cel7, Cel8), Cellulose synthase (celsyn), endo-xyloglucan transferase (Xytr), Expansin (NtExp1) and Glyceraldehyde-3-phosphate dehydrogenase (GAPDH)] was up regulated in *AtPME* transgenic lines (At-5) over WT (NTPH) while *AnPME* transgenic plants (An-4) also showed expression pattern similar to *AtPME* except Cel4. Transcript level of housekeeping genes like GADPH was almost unaltered in transgenic plants.

To observe the effect of over expression of PME on other important cell wall enzymes, we analysed the transcript level of certain important cell wall enzymes *viz*., endo-1,4-ß-glucanases (Cel2, Cel4, Cel5, Cel7, Cel8), Cellulose synthase (celsyn), endo-xyloglucan transferase (Xytr), Expansin (NtExp1) and Glyceraldehyde-3-phosphate dehydrogenase (GAPDH) by real time PCR (Figure 8). Transcript level of all analysed genes was up regulated in *AtPME* transgenic lines over WT. *AnPME* transgenic plants also showed expression pattern similar to *AtPME* except Cel4 which was slightly down regulated (1.3 fold). Transcript level of Cel5, Celsyn and NtExp1 was drastically increased in both types of transgenic plants. Transcript level up-regulation of these genes was 25, 37 and 35 folds in *AnPME* plants, and 29, 29 and 30 folds in *AtPME* plants, respectively. mRNA level of Cel7 (*AnPME*, 6.5 fold; *AtPME*, 3.5 fold) and Xytr (*AnPME*, 6.8 fold; *AtPME*, 5.3 fold) were also significantly increased in both types of transgenic plants. Cel2 was 1.3 and 1.9 fold while Cel8 was 1.1 and 1.9 fold up-regulated in *AnPME* and *AtPME* transgenic lines, respectively. Transcript level of housekeeping genes like GADPH was almost unaltered in transgenic plants (Figure 8). 

### Generation advancement and segregation of transgene in transgenic tobacco plants

T_0_ lines were self pollinated and seeds collected for T_1_ generation development. Total weight of collected seeds of each plant was measured individually and no significant yield penalty could be found in case of transgenic plants when compared to WT. T_1_ transgenic seeds were germinated and selected on antibiotic. T_1_ transgenic seeds showed average segregation ratio of ~1:2.7 (table S2). T_1_ transgenic lines were further confirmed by genomic DNA PCR, cDNA PCR, PME activity assay, and analysed for insect resistance. Segregation ratio of transgene in An-4 and At-5 plant was 2.12 and 2.33 respectively (table S2). 

T_1_ generation of An-4 plant showed 100% mortality of *H. armigera* and *S. litura* after 6 days respectively. While reduction in population of *M. persicae* and *B. tabaci* was 52%-98% and 25%-76% observed after 6 days (Figure S3A and S3B). T_1_ generation of At-5 plant also showed similar resistance pattern against all four insects. After 6 days, T_1_ plants caused mortality in the range of 56%-100% and 60%-88% of *H. armigera* and *S. litura* respectively. The reduction in the population of *M. persicae* and *B. tabaci* was 56%-94% and 33%-74% respectively (Figure S3A and S3B). Best performing T_1_ plants of selected lines (An-4 and At-5) were also tested against the voracious leaf feeder fourth instar larvae of *S. litura*. The larvae were unable to feed on these leaves resulting in their eventual death due to starvation (Figure S3C). 

## Discussion

Present study explored the enhanced methanol production in transgenic plants as a tool to achieve broad spectrum insect resistance. Methanol emission from plants is a natural process and it is not toxic to plants. Methanol is naturally produced and gets accumulated in the plant leaves, emitted through stomata [28,29]. Foliar sprays of methanol as 50% aqueous concentration increased growth and development in C_3_ crop plants [12] probably due to effective photosynthesis and more utilization of light energy [30]. 

Methanol is produced during demethylation of cell wall pectin directed by PME [10,28,31]. Silencing of *PME* gene in transgenic plants led to 70% reduction of methanol emissions [16]. Methanol emission has been correlated with pathogen resistance [11]. Increased emission of methanol in plants has been observed following herbivore attack; however, no direct evidences are available for methanol emission with insect resistance. This study demonstrated for the first time that methanol emission can be utilized to confer broad range insect resistance to plants. 

To increase the methanol production in plants, we developed PME over expressing transgenic tobacco plants. We selected PME from two different sources- (1) plant: *A. thaliana* (*AtPME*) and (2) fungi: *A. niger* (*AnPME*). We used *AtPME* because of its plant origin and expected to be easily expressed in transgenic plants. However, AtPME activity could be controlled by native plant inhibitors in the transgenic plants. The selected *AtPME* has *pme* encoding domain only (*pmei* domain was absent) hence PMEI level in transgenic plant should be same as WT plants. In case of AnPME, it has structurally different catalytic domain, hence plants PMEI cannot inhibit its activity due to structural incompatibility [17]. Therefore, AnPME was also alternatively used for development of transgenic plants. 

PMEs plays vital role in plant development; hence, we have critically examined growth, structural and physiological parameters of *AtPME* and *AnPME* transgenic plants. Transgenic plants showed normal growth pattern (Figure S1) and physiological structures were similar to WT plants (Figure 7). 

Methanol content in the leaves of transgenic plants was higher (~16 fold) as compared to control plants (Figure 4A) and the rate of methanol emission through stomata on leaves surface was also higher (~12.61 fold) than control plant (Figure 4B). Over expression of PMEs is reported to increase methanol production in plant cells [14]. The transgenic plants were expressing PME only but not PMEI, hence they had higher PME to PMEI ratio and more PME activity. This ultimately resulted into higher emission of methanol. Native PMEI of tobacco can only inhibit the AtPME to certain extent but not the AnPME due to the structural difference in catalytic domain [17]. Despite of some differences in PME activity (Figure 3A) in our best transgenic lines of both genes (At-5 and An-4), the amount of methanol recorded was similar (Figure 4A). This could be due to the high activity of AtPME in transgenic plants as compared to AnPME. Plant source of origin might also be responsible for the same. 

Our result showed that methanol content in transgenic plants leaves is ~16 fold higher than control plant but the rate of methanol emission through stomata is only ~12.61 fold higher, this is might be due to follicular emission of methanol. It shows extra amount of methanol generated by transgenic plant due to the over-expression of PME is emitted out by the plant. The amount of methanol content recorded from our best transgenic lines was still much lower than natural emission from several plants [21]. This might be the probable reason that we could not observe any deformity in plant growth and structural parameters in PME over expressing lines. The amount of methanol produced in transgenic plants may not be too high to affect plants cell environment. Moreover, knocking down of PME expression in transgenic tomato reportedly does not affect fruit yield or vegetative growth [32], suggesting that the level of methanol content may not affect the plant growth and development. Dwarfism has been reported in transgenic tobacco plants expressing PME which has been attributed to decreased expression level of genes involved in cell wall metabolism viz., endo-1,4-ß-glucanases (Cel2, Cel4, Cel5, Cel7, Cel8), Cellulose synthase (celsyn), endo-xyloglucan transferase (Xytr) and Expansin (NtExp1) [27]. In contrast, we did not find any difference in growth of transgenic and WT plants. We also observed very high expression (25-37 fold) of Cel5, Celsyn and NtExp1, moderate increase in Cel7 and Xytr (3.5-6.8 fold) and similar expression level of Cel2 and Cel8 genes in transgenics as compare to WT. The dwarfing of plants probably due to very high level of PME activity (2-3 fold increase) in young leaves of transgenic plants as compared to control [27]. We could only observe 39%- 42% increase in PME activity in transgenics over WT. 

Transgenic plants were evaluated for insect resistance against chewing (neonate larvae of *H. armigera* and *S. litura*) and sap sucking crop insects (adults of *M. persicae* and *B. tabaci*). Transgenic plants showed significant resistance against all the tested insects (Figure 5 and 6). Similar results were observed in insect bioassay on methanol incorporated artificial diet (table S3). In case of *M. persicae* the population increased continuously on WT plants during bioassay period. The percent decrease in population multiplication was calculated for transgenics and WT. In case of *B. tabaci*, population did not increase during the bioassay period. However, we could observe very good resistance in transgenic plants against all tested insects. 

Plants having higher degree of pectin methylation are known for resistance to pathogens [33,34], whereas the methanol emission by pectin demethylation is also known for increased resistance against plant pathogens [35]. Therefore, we evaluated transgenic plants for susceptibility against plant pathogenic fungi *Alternaria alternata* and bacteria *Pseudomonas syringae* pv. *maculicola*. Both WT and transgenic plants showed similar susceptibility (Figure S4). There is possibility that over expression of PMEs may enhance susceptibility of plants to viral infection, since PMEs are known to promote the movement and dispersal of tobacco mosaic virus [36,37]. However the PME over expression plants being highly resistant to insect vector (aphids and whiteflies) should effectively prevent virus spreading. 

PMEs are also known to play vital role in pollen tube development [38]. Hence, we compared pollen viability and germination of WT and transgenic plants which were found similar (Figure 7D and E). 

Insect resistant Bt-transgenic plants have greatly contributed towards reduction in global pesticide consumption and reduced loss of crop yields due to insect attack. Presently, a number of Bt-containing products are in the market of United States, China, India and other countries. Bt-technology has increased the productivity of crops like cotton and corn substantially by controlling the major insects of orders lepidoptera and coleoptera [39]. However Bt-transgenics are ineffective against sap sucking insects such as aphids and whiteflies [40]. There is a concomitant increase in the population of minor pests like whiteflies, aphids, leafhoppers on Bt-transgenic crops [4–6]. Sap sucking pests have become more problematic in Bt-cotton fields than in conventional fields [41]. Whiteflies and aphids are the most potent vectors for transmission of viruses from plant to plant. In United States, loss due to whiteflies on cotton, tomato and vegetable crops was estimated to be more than $500 million [42]. Therefore, effective proteins against sap sucking pests are in great demand for pyramiding with Bt genes in order to obtain broad spectrum insect resistance in transgenic plants. Proteins like lectins (agglutinins) can provide some degree of protection against sap sucking pests but needs to be expressed at very high concentrations. Recently a transplastomic tobacco plants expressing *Pinellia ternata* agglutinin showed broad spectrum insect resistance [43]. Transplastomic expression yields very high level of expression (9.27% of TSP), which is not possible to achieve in stable transgenic lines by nuclear transformation. Very high level of expression of foreign protein also cause “yield penalties” in the host plants under normal growth conditions due to side effects of over-expression of the transgene [44,45]. 

Production of methanol in transgenic plants can provide wide range insect resistance without compromising the yield and health of plants. Over-expressed PME is able to produce enough amount of methanol required for providing protection against wide range of insect pests. Insect inducible expression of PME will further reduce environmental risk. Therefore, PME can be used for genetic transformation of crop plants for minimizing the crop loss due to insect pests without any yield penalties and health hazards.

## Supporting Information

Figure S1 **Bioassay against whiteflies using leaf discs.** (A) Agar-agar pored in caps of bioassay vial. (B) Fitting of bioassay vial containing whiteflies to caps having leaf discs placed on agar-agar. (C) Bioassay tubes containing leaf dicks and whiteflies. (D) Magnified image of C.
(TIF)

Figure S2 **Growth and development of transgenic and control plants.** (A and B ) comparisons of growth of transgenics (An-4 of AnPME and At-5 line of AtPME) and control plants. © table showing various average of plant growth parameters. (ANOVA, F* test, non significant). .
(TIF)

Figure S3 **Evaluation of T_1_ plants for insect resistance.** (A) Bioassay against neonate larvae of *H. armigera* and *S. litura*. B, *In-planta* bioassay against *M. persicae* and *B. tabaci*. (C) Bioassay of selected plants of T_1_ against fourth instar larvae of *S. liture*. Larvae could not feed on transgenic plants (T1- T2 & T3), while control leaves (C) were completely fed within 24 hours .
(TIF)

Figure S4 **Leaves of transgenic plant and control were infected with plant pathogenic fungi *Alternariaalternata* and bacteria *Pseudomonas syringae* pv. *maculicola* strain ES4326 (PsmES4326).** Fungus and bacteria both were grown on transgenic as well as control plant and does not have any significant difference on susceptibility.
(TIF)

Table S1 **Calculation of methanol quantification in transpirated water through stomata.**
(DOCX)

Table S2 **Segregation Ratio of transgene in T_1_ generation on kanamycin selection.**
(DOC)

Table S3 **Insect bioassay of *Helicoverpa armigera* and *Spodoptera litura* on aritificial diet with different concentration of methanol.**
(DOCX)
