# Supplementary figures and images for: Enhanced Methanol Production in Plants Provides Broad Spectrum Insect Resistance
Source: PLoS One. 2013 Nov 5;8(11):e79664. doi: 10.1371/journal.pone.0079664 (PMC3818224; doi:10.1371/journal.pone.0079664)

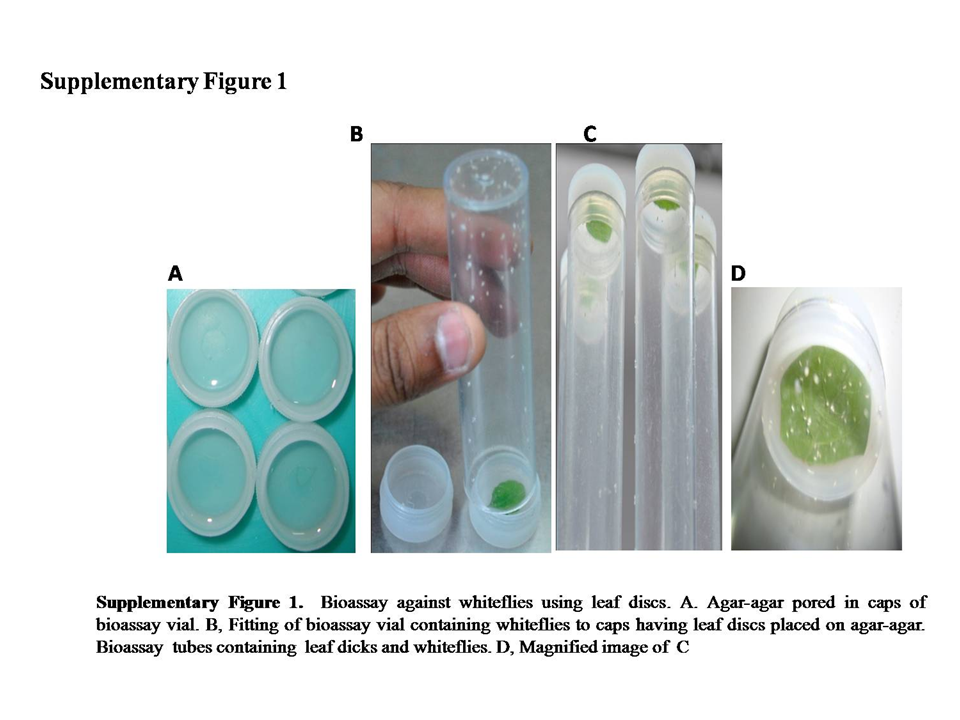

Supplement: Figure S1 — Bioassay against whiteflies using leaf discs. (A) Agar-agar pored in caps of bioassay vial. (B) Fitting of bioassay vial containing whiteflies to caps having leaf discs placed on agar-agar. (C) Bioassay tubes containing leaf dicks and whiteflies. (D) Magnified image of C. (TIF) [file pone.0079664.s001.tif]

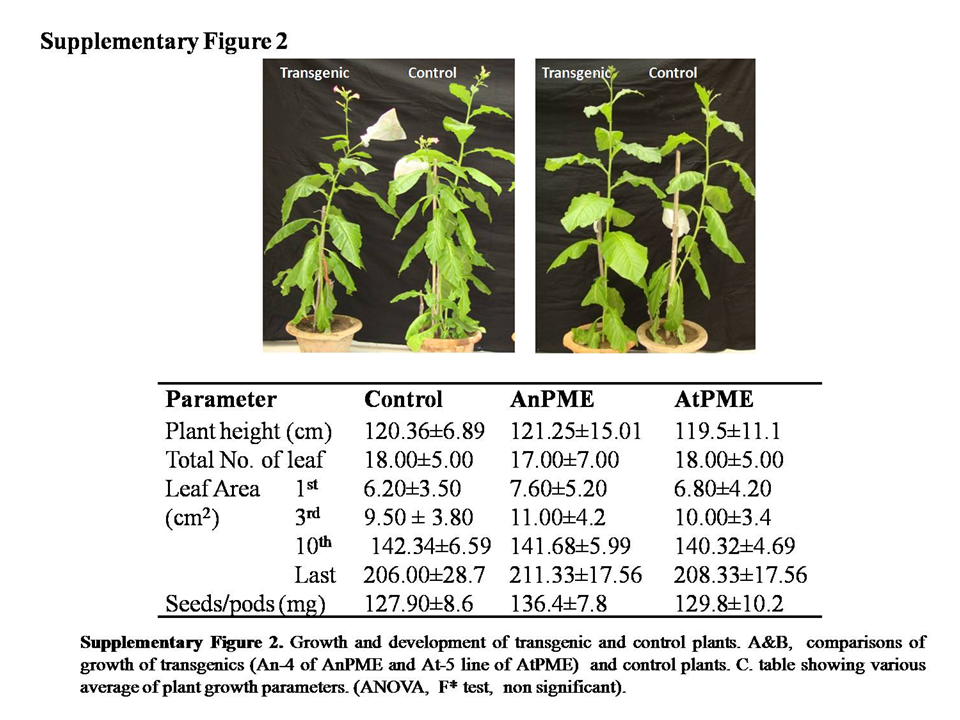

Supplement: Figure S2 — Growth and development of transgenic and control plants. (A and B ) comparisons of growth of transgenics (An-4 of AnPME and At-5 line of AtPME) and control plants. © table showing various average of plant growth parameters. (ANOVA, F* test, non significant). . (TIF) [file pone.0079664.s002.tif]

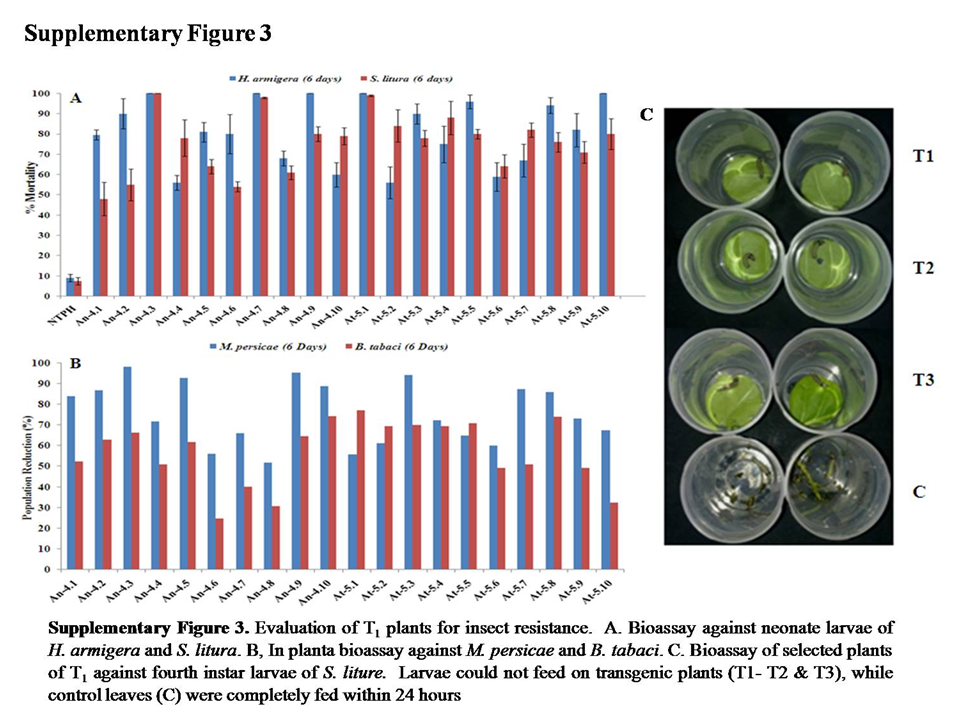

Supplement: Figure S3 — Evaluation of T1 plants for insect resistance. (A) Bioassay against neonate larvae of H. armigera and S. litura. B, In-planta bioassay against M. persicae and B. tabaci. (C) Bioassay of selected plants of T1 against fourth instar larvae of S. liture. Larvae could not feed on transgenic plants (T1- T2 & T3), while control leaves (C) were completely fed within 24 hours . (TIF) [file pone.0079664.s003.tif]

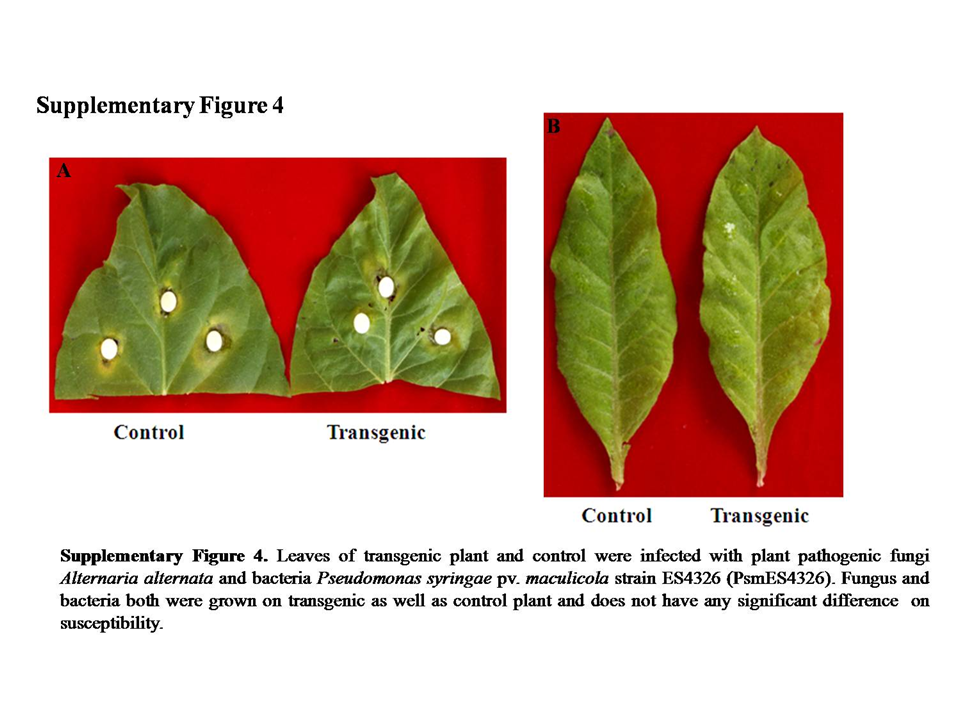

Supplement: Figure S4 — Leaves of transgenic plant and control were infected with plant pathogenic fungi Alternariaalternata and bacteria Pseudomonas syringae pv. maculicola strain ES4326 (PsmES4326). Fungus and bacteria both were grown on transgenic as well as control plant and does not have any significant difference on susceptibility. (TIF) [file pone.0079664.s004.tif]
