# Supplementary material for: Enhanced Methanol Production in Plants Provides Broad Spectrum Insect Resistance
Source: PLoS One. 2013 Nov 5;8(11):e79664. doi: 10.1371/journal.pone.0079664 (PMC3818224; doi:10.1371/journal.pone.0079664)
Supplement: Table S1 — Calculation of methanol quantification in transpirated water through stomata. (DOCX) [file pone.0079664.s005.docx]

Supplementary Table 1: Calculation of methanol quantification in transpirated water through stomata.

| Plants | Leaf Surface Area (cm^2^) | Total Incubation Time (min) | Volume of Total Transpirated Water (µl) | Total Emitted Methanol (µmole) | Rate of Methanol Emission  (µmole min^-1^cm^-2^) |
| --- | --- | --- | --- | --- | --- |
| NTPH | 12.92±3.17 | 120 | 367±4 | 614.1 | 0.39±0.01 |
| An | 16.12±4.55 | 120 | 375±6 | 8442.5 | 4.36±0.21 |
| At | 14.66±3.78 | 120 | 370±8 | 8667 | 4.92±0.21 |
