# Supplementary material for: Enhanced Methanol Production in Plants Provides Broad Spectrum Insect Resistance
Source: PLoS One. 2013 Nov 5;8(11):e79664. doi: 10.1371/journal.pone.0079664 (PMC3818224; doi:10.1371/journal.pone.0079664)
Supplement: Table S2 — Segregation Ratio of transgene in T1 generation on kanamycin selection. (DOC) [file pone.0079664.s006.doc]

| Plants | Total no. of seeds shown | Total no. of seeds grow on selection of Kanamycin (300 mg/l) | Segregation Ratio in T_1_ generation |
| --- | --- | --- | --- |
| NTPH | 100 | 5 | ----- |
| An-1 | 100 | 70 | 2.33 |
| An-2 | 100 | 71 | 2.44 |
| An-3 | 100 | 76 | 3.16 |
| An-4 | 100 | 68 | 2.12 |
| An-5 | 100 | 71 | 2.44 |
| At-1 | 100 | 78 | 3.54 |
| At-2 | 100 | 63 | 1.70 |
| At-3 | 100 | 70 | 3.76 |
| At-4 | 100 | 78 | 3.54 |
| At-5 | 100 | 79 | 2.33 |

Supplementary Table 2: Segregation Ratio of transgene in T_1_ generation on kanamycin selection.
