# Supplementary material for: Enhanced Methanol Production in Plants Provides Broad Spectrum Insect Resistance
Source: PLoS One. 2013 Nov 5;8(11):e79664. doi: 10.1371/journal.pone.0079664 (PMC3818224; doi:10.1371/journal.pone.0079664)
Supplement: Table S3 — Insect bioassay of Helicoverpa armigera and Spodoptera litura on aritificial diet with different concentration of methanol. (DOCX) [file pone.0079664.s007.docx]

Supplementary Table 3: Insect bioassay of *Helicoverpa armigera* and *Spodoptera litura* on aritificial diet with different concentration of methanol. Result indicated that almost 100 % mortality was caused at 30 µl/g concentration in both insect. Below it sever growth retardation was observed. Size and weight of surviving larvae on methanol diet was very much low with compared to larvae on normal diet.

| Insect bioassay on aritificial diet with different concentration of methanol | | | | | | |
| --- | --- | --- | --- | --- | --- | --- |
| Methanol Concentration (µl/g of diet) | *Helicoverpa armigera* | | | *Spodoptera litura* | | |
|  | Percentage Mortality  (Average weight of larvae after 72 hrs ) | | | | | |
|  | 24 hrs | 48 hrs | 72 hrs | 24 hrs | 48 hrs | 72 hrs |
| 10 µl/g | 10 % | 10 % | 35 %  (1.8±0.3 g) | 10 % | 15 % | 25 %  (0.9±0.3 g) |
| 20 µl/g | 10 % | 25 % | 40 %  (0.7±0.2 g) | 15 % | 35 % | 60 %  (0.2±0.1 g) |
| 30 µl/g | 65 % | 90 % | 100 %  (0.0±0.0 g) | 40 % | 55 % | 95 %  (0.1±0.0 g) |
| 40 µl/g | 100 % | 100 % | 100 %  (0.0±0.0 g) | 100 % | 100 % | 100 %  (0.0±0.0 g) |
| 50 µl/g | 100 % | 100 % | 100 %  (0.0±0.0 g) | 100 % | 100 % | 100 %  (0.0±0.0 g) |
| Diet without methanol | 0 % | 0 % | 0 %  (2.1±0.5 g) | 0 % | 0 % | 0 %  (1.5±0.2 g) |

In the case of sapsucking insect (*Myzus persicae* and *Bemisia tabaci*), we found that each time all the insect were died with in 24 hrs at very low concentration of methanol on liquid artificial dite (10 µl/ml) and no mortality was ocuured in control dite. Experiment was performed as described by Upadhyay et al. 2011.

Reference:

1. Upadhyay SK, Chandrashekar K, Thakur N, Verma PC, Borgio JF, Singh PK, Tuli R (2011) RNA interference for the control of whiteflies (Bemisia tabaci) by oral route. J Biosci. 36:153-61.
